# Aminated Polyethylene Terephthalate (PET) Nanofibers for the Selective Removal of Pb(II) from Polluted Water

**DOI:** 10.3390/ma10121352

**Published:** 2017-11-24

**Authors:** Diego Morillo Martín, Mohamed Magdi Ahmed, Mónica Rodríguez, María A. García, Mirko Faccini

**Affiliations:** 1 Leitat Technological Center, C/Pallars, 179-185, 08005 Barcelona, Spain; dmorillo@leitat.org (D.M.M.); mohmagdi@yahoo.com (M.M.A.); 2 Centro de Excelencia en Nanotecnología Leitat, Fundación Leitat Chile, Mariano Sánchez Fontecilla 310, 701-D, Las Condes, Santiago 8320000, Chile; mrodriguez@fundacionleitat.cl (M.R.) magarcia@fundacionleitat.cl (M.A.G.)

**Keywords:** poly(ethylene terephthalate) (PET), surface modification, electrospinning, adsorption nanofibers, wastewater treatment, water remediation, heavy metals pollution, lead removal

## Abstract

Electrospun nanofibers have been successfully applied to remove toxic and carcinogenic contaminants such as heavy metals from polluted water. In this study, an efficient adsorbent based on poly(ethylene terephthalate) (PET) nanofibers was developed following a cheap, versatile and scalable process. PET nanofibers were first produced by electrospinning, and their surface was chemically functionalized using a simple aminolysis process. The capacity of the resulting material to adsorb Pb(II) from synthetic solutions was evaluated as a function of the contact time, pH, and initial metal ion concentration. The adsorbent system presented a quick kinetic adsorption, reaching an extremely high maximum adsorption capacity of about 50 millimol (mmol) of Pb(II) per gram of adsorbent system after just 30 min. Moreover, the effect of competing metal ions, such as Ni(II), Cd(II) and Cu(II), was studied at different molar ratios. Finally, when tested in continuous flow mode, aminated PET (APET) nanofibers were able to remove 97% of Pb(II) ions in solution, demonstrating their potential for the remediation of heavy metal-contaminated water.

## 1. Introduction

The pollution of water by heavy metals has been considered a major threat to the environment and living organisms. Metals such as copper, nickel, lead, and cadmium can be harmful to human health even at very low concentrations, as they tend to accumulate, thereby physiologically and/or neurologically affecting exposed individuals [1].

A wide variety of techniques, including physico-chemical methods, chemical precipitations, coagulation and flocculation, electrochemical treatments, ion exchange, membrane filtration, electrodialysis, and biological methods, are used to treat polluted water [2]. Among these, adsorption is commonly regarded as a simple, effective, and economical method for this application [3]. Various types of adsorbents, such as activated carbons, natural zeolites, biosorbents or chelating materials, have been studied as adsorbent systems to extract metal ions from aqueous solutions. The properties of these adsorbents depend on their surface-area-to-volume ratio and on the functional groups on their surfaces [4].

The adaptation of nanotechnology to process engineering for the improvement of water quality using nanosorbents, nanocatalysts [5,6], nanoparticles [7,8,9,10], and nanostructured membranes [11,12,13] among other nanotechnology-based products has demonstrated applicability for these alternatives in water treatment. Nanoadsorbents, such as nanobeads, nanocomposites [14,15,16,17], magnetic nano-adsorbents [18,19,20], and nanofiber matrices [21,22,23,24,25,26], are applicable in the detection and removal of various pollutants. In particular, nanofibers are a subject of intensive research because of their large surface-area-to-volume ratio, flexibility in surface functionality, and mechanical properties such as stiffness and tensile strength. Nanofibers also demonstrate the capacity to be formed into a variety of shapes, and they can be produced from a wide range of organic and inorganic polymers and ceramics [27].

Electrospinning is a powerful technique in the production of nanofiber-based technology. This technique is a straightforward and versatile way to generate electrospun fibers with different morphologies on the nano- to micro-scale [28]. Recent increases in studies and patents, as well as a broadening range of chemical, medical, electrical, and environmental applications, demonstrate the global interest in nanofibers [29]. Nanofiber chemical modifications can be performed over electrospun nanofibers by introducing functional groups with specific functionality to improve the adsorption properties of the nanofibers. These modified nanofibers can also be applied as an adsorbent in wastewater treatment [30,31,32,33,34].

Poly(ethylene terephthalate) (PET) is one of the most consumed thermoplastics in the world and is used for a variety of purposes with different end-product applications such as films, non-woven textiles, containers, and engineering plastics. The potential usefulness of PET exists for other applications such as protective clothing, filtration devices, membranes, vascular grafts, and tissue scaffolding. These products are clearly related to PET´s unique properties, such as its glass transition temperature (T_g_), melting temperature (T_m_), tensile strength, tensile elongation, and excellent barrier properties, among others [35].

Previous studies show that PET plays a remarkable role as a filter for selective lead removal from aqueous solutions. Coskun et al. [36] prepared adsorbent fibers by the graft copolymerization of acrylamide (AAm) onto PET fibers and examined the adsorption properties of Pb(II) ions from aqueous solutions by the fibers using a batch equilibration technique. Temoçin et al. [37] studied the adsorption behavior of 4-vinyl pyridine- and 2-hydroxyethylmethacrylate-grafted PET fibers toward the Hg(II) and Pb(II) ions in aqueous solutions by a batch equilibration technique. Ahmad et al. [38] prepared a fibrous adsorbent by grafting acrylic acid/acrylamide (AA/AAm) comonomers onto PET fibers. Niu et al. [39] fabricated a chitosan-modified PET composite adsorbent using a dip-padding method for the removal of the toxic metal ions Zn(II), Cu(II), and Pb(II) from aqueous solutions. Meng et al. [40] synthesized fibers by the graft copolymerization of acrylic acid (AA) onto PET. Moreover, modification of the grafted PET fiber (PET–AA) was done by changing the carboxyl group into acrylamino and amino groups through a reaction with ethylenediamine (EDA). The adsorption isotherm indicated that the grafted PET fiber had a high equilibrium adsorption capacity for Pb(II).

In this work, a straightforward and industrially scalable adsorbent material based on electrospun PET nanofibers was developed to remove heavy metals from polluted water. To this end, PET nanofibers with diameters of around 500 nm were functionalized using EDA as an aminolysis reagent. The influence of the degree of surface modification on mat degradation and adsorption properties was first studied in batch mode. The optimal nanostructured adsorbent was placed in a cartridge and tested in the continuous-flow operation mode. This demonstrated that aminated PET nanofiber mats are a powerful tool for the remediation of polluted waters.

## 2. Materials and Methods

### 2.1. Reactants and Solvents

PET pellets, dichloromethane (DCM; reagent grade), trifluoroacetic acid (TFA) and tetrabutyl ammonium bromide (TBAB) were used to prepare polymeric solutions for the electrospinning process. EDA (99 % purity) was used in the aminolysis process. Absolute ethanol was used as the solvent in a Kaiser test, and lead nitrate (Pb(NO_3_)_2_), copper chloride (CuCl_2_·2H_2_O), nickel chloride (NiCl_2_·6H_2_O), and cadmium nitrate (Cd(NO_3_)_2_·4H_2_O) were used for heavy metal synthetic solutions.

### 2.2. Electrospinning Process

Solutions for electrospinning were prepared by dissolving 15–20 wt % PET pellets in a 1:1 solvent mixture of DCM and TFA. In addition, 0.1–0.2 wt % TBAB was added to increase the electrical conductivity. All polymer solutions were mixed by using a magnetic stirrer for a sufficient time until they became homogeneous (6–8 h). The viscosity and electrical conductivity were measured by a digital viscometer (DV-E, Brookfield Co., Ringgold, GA, USA) and an electric conductivity meter (CRISON EC–meter BASIC, Barcelona, Spain), respectively, at 25 °C.

The PET solutions were electrospun by using a commercially available electrospinning setup (model NF-103, MECC Co., Ltd., Fukuoka, Japan) equipped with a rotary collector drum (200 rpm). Plastic syringes fitted with metal needles (GA25; 0.25 mm ID × 38.1 mm length) were used as electrospinning nozzles, and a non-woven polyester textile was employed as a support for the electrospun PET nanofibers. The detailed operating conditions employed to collect the nanofibers are reported in Table 1.

### 2.3. Aminolysis of PET Nanofiber Mats

Aminated PET (APET) nanofiber mats were generated by immersing the electrospun PET nanofiber mats (4–10 mg) in 100 mL of an aqueous EDA solution with concentrations of 2.5, 5 and 10 wt %, at 40 °C and over contact times of 15 and 60 min.

### 2.4. Characterization

#### 2.4.1. Scanning Electron Microscopy

The morphology of both the electrospun PET and APET nanofiber mats were observed through the use of a scanning electron microscope (SEM; Jeol, JSM 6010LV, Peabody, MA, USA). Each specimen was coated with a thin layer of gold. Diameters of no less than 50 individual fiber segments for a given fiber mat specimen were measured in the SEM images using InTouchScope software (Jeol, Peabody, MA, USA), from which the average values were calculated.

#### 2.4.2. Elemental Analysis

Elemental analysis (EuroVector, EuroEA3000, Redavalle, Italy) was carried out to analyze C, H, and N elements. The mass fraction for oxygen was calculated relative to the theoretical mass of the APET nanofiber mats and the elemental analysis results.

#### 2.4.3. Kaiser Test

The Kaiser test, a colorimetric method, was used to qualitatively determine primary amine groups’ presence on the electrospun PET nanofiber surface. Three drops of the solutions 80% phenol in ethanol, 65% KCN in H_2_O/pyridine, and 6% ninhydrin in ethanol were added to the APET nanofiber mat samples and mixed. After mixing, a reaction between ninhydrin with free primary amines was conducted at 120 °C for 5 min, generating an intense blue color [41].

#### 2.4.4. Attenuated Total Reflectance–Fourier Transform Infrared Spectrometry (ATR–FTIR)

A Fourier transform infrared spectrometer (SHIMADZU, IRAffinity-1, Chiyoda-ku, Tokyo, Japan) hyphenated to an attenuated total reflectance (ATR) system, operating at a resolution of 4 cm^−1^ and a wavenumber range from 4000 to 400 cm^−1^, was used to determine the functional groups of the neat and modified electrospun PET fiber mats.

#### 2.4.5. Inductively Coupled Plasma Mass Spectroscopy (ICP-MS)

After each adsorption experiment, the nanofiber sample was removed from the solution and the remaining metal ion content was quantified by inductively coupled plasma mass spectroscopy (ICP-MS; Agilent, ICP-MS 7500, Santa Clara, CA, USA).

### 2.5. Adsorption Behavior in Batch Mode

The amount of metal ions adsorbed onto each nanofiber mat specimen (adsorption capacity, *q,* in mmol g^−1^) was calculated on the basis of the following Equation (1):

(1) q=(C0−Ce)VMMads

where *C_o_* and *C_e_* are the initial and the equilibrium concentrations of the metal ion (mg L^−1^), *V* is the volume of the metal ion solution (L), *M* is the atomic weight of the metal ion (mg mmol^−1^), and *M_ads_* is the weight of the adsorbent (g).

The experiments were carried out by mixing 40 mL of Pb(II) solution with a constant amount of APET nanofiber mats (5–15 mg). All samples were shaken in a vibratory shaker and the pH of the solutions was adjusted using 1.0 M HNO_3_ or 1.0 M NaOH standardized solutions. To investigate the effect of the contact time, experiments were performed at an initial Pb(II) concentration of 100 mg L^−1^ and at pH 6. Sample uptake was carried out at 1, 5, 10, 20, 30, 60, 120, 180 and 240 min. To investigate the pH effect, Pb(II) solutions (100 mg L^−1^) at different pH values (close to 2, 4, 5, 6, 7, 8, 10 and 12) were prepared. The contact time for each adsorption experiment was 30 min. To investigate the effect of the ion concentration, the Pb(II) solutions were prepared in a wide concentration range (1, 5, 10, 50, 100, 250, 500 and 1000 mg L^−1^) at pH 8, and the experiments were carried out with 30 min of contact time (where the maximum adsorption was reached previously). To determine the selective adsorption of the APET nanofibers, solutions with different molar ratios of the target ion Pb(II) to the common interfering ions Cu(II), Ni(II), and Cd(II) were prepared at ratios of 1:0, 1:1, and 1:2. The experiments were carried out under the experimental conditions giving the highest adsorption capacity, at pH 8, with 30 min of contact time and 250 mgL^−1^ Pb(II) concentration.

### 2.6. Metal Adsorption in Continuous Mode

Once the optimal conditions for Pb(II) adsorption on the APET nanofibers in the batch mode were determined, adsorption experiments in the continuous mode were performed to assess the behavior of the adsorbent system under real working conditions. For this purpose, a cartridge was designed consisting of 10 disks of the developed selective membrane (approximately 150 mg of material) spaced with membranes of commercial polypropylene and glass wool (see Figure S1, Supplementary Materials). With the aid of a peristaltic pump (ISMATEC, model MCP-Z Standard), 1 L of 5 mg L^−1^ Pb(II) synthetic solution at pH 8 was recirculated throughout the cartridge with a pumping rate of 20 L/h for 6 h. Sampling was performed at the start time, again after 1 min, every 5 min until 30 min had passed, and then every 30 min until 6 h had passed.

## 3. Results and Discussion

### 3.1. Electrospun PET Nanofibers

It is well known that the morphology of electrospun fibers depends on various processing parameters and environmental conditions. Several parameters such as concentration, conductivity, and the viscosity of the solutions significantly affect the electrospinning process and thus also the nanofiber morphology. In order to obtain a stable jet and a homogeneous process, solutions with different PET and TBAB concentrations were prepared, and their viscosity and conductivity were measured, as shown in Table 2. Generally, the polymer concentration in the electrospinning solutions presents an important effect over both viscosity and conductivity, and it is necessary to control these parameters to reach a stable and homogeneous process. In the current study, when the PET concentration was reduced from 20 wt % (PET-1) to 15 wt % (PET-2), while keeping the TBAB salt concentration constant at 0.1 wt %, the viscosity decreased from 298.5 to 170.4 cP. The conductivity, however, increased from 10.7 to 46.8 µS/cm as an effect of the presence of more TFA molecules in the solution. This fact had an effect on the fiber diameter, which decreased drastically from 3 µm to 724 nm. On the other hand, raising the TBAB concentration to 0.2 wt % (PET-3) resulted in an increase of the solution’s conductivity from 46.8 to 126.2 µS/cm, improving the stability of the electrospinning process and reaching a smaller nanofiber diameter (488 nm) with a narrow range [42].

Different operating conditions for each solution were set to produce nanofibers in a continuous and stable electrospinning process. In general, the electrical conductivity is inversely proportional to the electric field (voltage/distance) for fiber formation. For solution PET-1, which had the highest viscosity, the electrospinning process was sufficiently stable, and a relatively low voltage of 25 kV had to be applied to form fibers. Meanwhile, solution PET-3, in spite of its lower viscosity, required a higher electric field to be electrospun (Table S1, Supplementary Materials).

**Figure 1:**
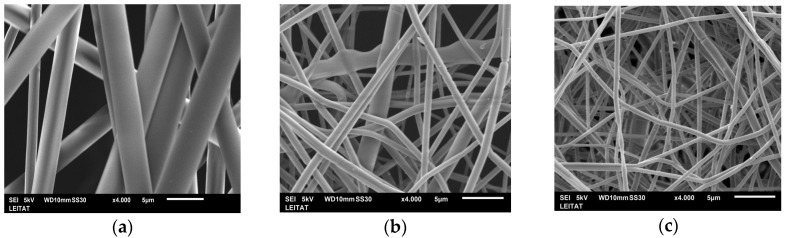
Scanning electron microscope (SEM) images of electrospun poly(ethylene terephthalate) (PET) nanofiber mats synthesized with different solutions: (**a**) PET-1; (**b**) PET-2; (**c**) PET-3.

In order to observe the effect of the PET and TBAB concentrations on the nanofiber diameters, we analyzed SEM images of electrospun PET nanofibers produced by electrospinning different polymer solutions, as shown in Figure 1.

In all cases, the cross-sections of the PET nanofibers were round and their surfaces were smooth. The PET concentration was the parameter with the strongest influence on the electrospinnability of the solution, resulting in larger nanofiber diameters as the polymer concentration increased. Although PET fibers from the 20 wt % solution (PET-1) were collected with a stable process, their diameter was in the micrometer range, at approximately 3.0 ± 0.5 µm. Decreasing the polymer concentration to 15 wt % while keeping constant the TBAB wt % (PET-2) resulted in a reduction in the average fiber diameter size to 724 ± 125 nm.

Raising the conductivity of the PET solution from 0.1 to 0.2 wt % TBAB (PET-3) while keeping the PET concentration at 15 wt % achieved a more stable jet. Consequently, smaller and more homogeneous nanofibers with an average diameter size of 488 ± 61 nm were electrospun. High conductivity gives better stability to the electrospinning process. At a lower viscosity, there is less chain entanglement and more chain mobility, resulting in more extension during spinning, which altogether produces thinner fibers [43,44].

Considering the diameters of the nanofibers obtained at different polymer concentrations, the PET solution at 15 and 0.2 wt % TBAB (PET-3) was selected to continue the subsequent aminolysis processes.

### 3.2. Aminated PET Nanofiber Mats

**Scheme 1:**
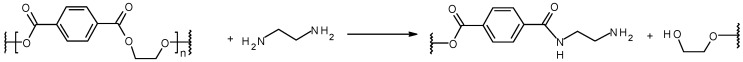
Mechanism of poly(ethylene terephthalate) (PET) aminolysis process.

A primary amine group was introduced to the electrospun PET nanofiber via the aminolysis process using chemical grafting with EDA. This chemical treatment is industrially scalable and highly versatile, as it can be applied using a wide variety of reagents with free primary amine groups. As a result of the aminolysis reaction between the amino-based molecule and PET surface, ester-links (O=C–O) of PET are replaced with covalent amide bonds (O=C–N), following the chemical equation shown in Scheme 1 [45].

The Kaiser test is a qualitative test that determines the presence or absence of free primary amino groups [41]. The test is based on the reaction of ninhydrin with primary amines, which gives a characteristic dark blue color. The test requires minimal amounts of material and is completed within a few minutes. Hence, the Kaiser test was employed as a fast screening method to confirm the presence of amine groups on PET nanofiber mat surfaces, thereby assessing the efficiency of the aminolysis process.

Initially, aminolysis was performed at room temperature with EDA concentrations of 10 and 40 wt %, however, no blue color was visible after Kaiser test, indicating that the modification was either too low or simply non-existent at such conditions (Figure S2, Supplementary Materials). Therefore, the temperature was slightly increased. The aminolysis process was carried out at 40 °C and varying the EDA concentration in ethanol between 10 and 60 wt %. Applying EDA concentrations above 20 wt % during the aminolysis process resulted in a degradation of the nanofiber mats, which gradually lost their consistency as the EDA concentration increased. Hence, such high EDA concentrations were discarded, and the functionalization studies were continued using EDA concentrations between 2.5 and 10 wt % and contact times of 15 and 60 min, as listed in Table 3.

**Figure 2:**
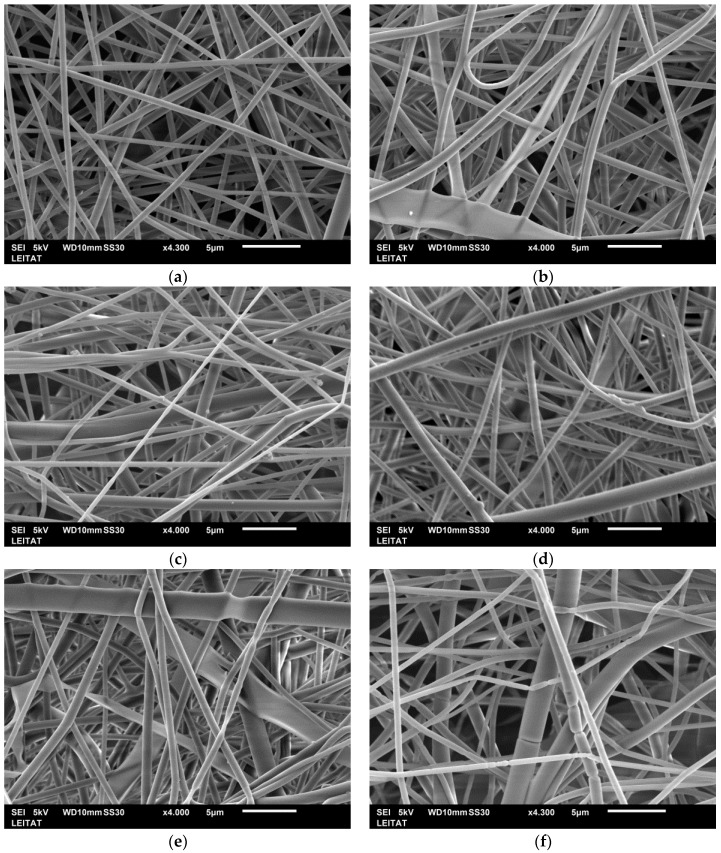
Representative scanning electron microscope (SEM) images of aminated poly(ethylene terephthalate (APET) electrospun fiber mats at different aminolysis conditions over 60 min: (**a**) APET-1, (**b**) APET-3, and (**c**) APET-5; and over 15 min: (**d**) APET-2, (**e**) APET-4, and (**f**) APET-6.

APET nanofiber samples (Figure 2) were analyzed with a SEM to observe the effect of the EDA concentration on the fiber morphology. For all the samples, no apparent damage was observed; the mats retained good consistency after the reaction, and APET nanofiber diameters were larger than those of the electrospun PET nanofibers. However, it is noteworthy that the aminolysis, which occurred with shorter contact times (15 min), presented higher diameters of the APET nanofibers (around 40–50 nm) compared to those with longer contact times (60 min). Thus, as the contact time increased, the fiber degradation became greater [46].

Elemental analysis was employed to quantify the degree of functionalization of the APET nanofibers, which was calculated as milligrams of EDA per gram of APET fibers. As shown in Table 2, a shorter contact time (15 m) resulted in a more efficient aminolysis process, with the percentages of nitrogen in the APET fibers ranging between 4.97% and 6.73%, which corresponded to 0.11–0.14 mg EDA/g of fibers. On the contrary, a longer contact resulted in a low modification degree of only about 0.2 mg EDA/g of fibers, regardless of the EDA concentration employed. This fact can be explained by the degradation observed in the SEM images presented above. At longer contact times, the electrospun PET nanofiber surface was degraded, and a certain amount of surface functional groups with amide groups were removed and released into the reaction media. When the nanofiber mats were left reacting for longer with EDA, a certain degree of degradation occurred that reduced the active centers available on the electrospun nanofibers, thus resulting in a poor surface functionalization. On the other hand, at shorter contact times, the surface degradation was negligible and the PET chemical grafting was favored.

These quantitative results fit well with the qualitative results obtained from Kaiser testing, as the color intensity was stronger for 15 m of contact time (APET-2, APET-4 and APET-6) and decreased when the contact time increased (Figure S3, Supplementary Materials). These results confirm that a colorimetric test is a useful tool for a quick estimation of the degree of aminolysis.

Regarding the EDA concentration, the modification process with 5 wt % EDA (APET-4) over 15 min of contact time presented the highest degree of surface functionalization at 0.14 mg EDA/g of APET nanofibers.

**Figure 3:**
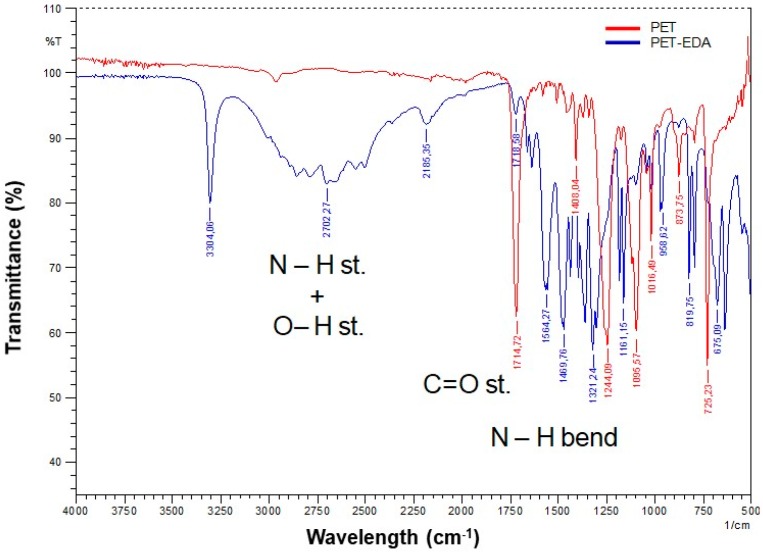
ATR–FTIR spectra of the neat electrospun poly(ethylene terephthalate) (PET) fiber mat and the aminated PET (APET) electrospun fiber mat.

Through ATR–FTIR spectroscopy, functional groups of pristine and modified electrospun PET fiber mats were determined, as shown in Figure 3, demonstrating that the aminolysis process was successfully achieved. For PET mats, the carbonyl stretch was lowered to 1714 cm^−1^. A strong peak at 1244 cm^−1^ was observed at the symmetric C–C–H stretch that involves the carbon in the aromatic ring. The aromatic wag C–H was also affected by the carbonyl group and shifted down to 725 cm^−1^. For the APET fiber mats, a medium peak was observed at 3304 cm^−1^, and a broad peak was observed at 2702 cm^−1^. These peaks corresponded to the NH_2_ stretch and the N–H wag, respectively.

The conditions that provided a higher degree of functionalization (APET-4) were then used to make modified membranes to perform Pb(II) adsorption tests and to determine the main parameters of the process, as well to determine the selectivity against other metal ions.

### 3.3. Adsorption Experiments in Batch Mode

The adsorption capacity of APET to remove Pb(II) from contaminated water was evaluated in order to determine the optimal conditions for the adsorption process, including the contact time, initial pH, initial concentration, and selectivity towards metal ions.

#### 3.3.1. Effect of Contact Time in the Adsorption Process

**Figure 4:**
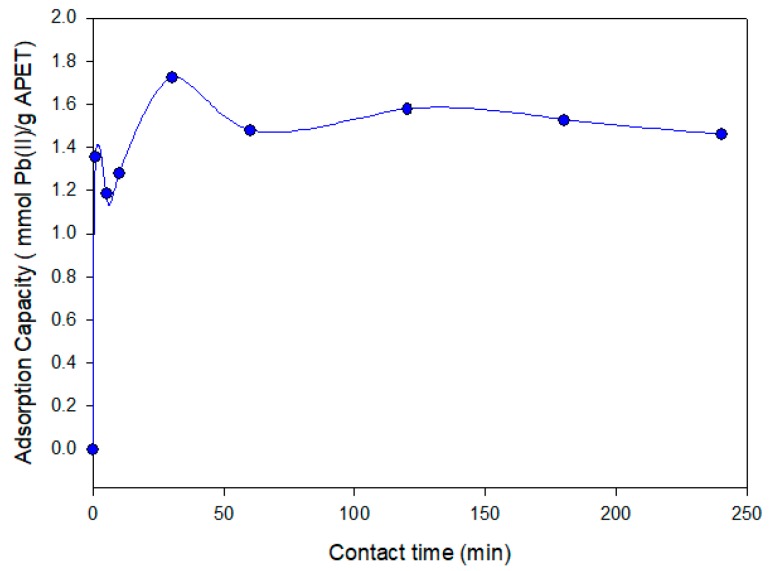
Effect of contact time on the Pb(II) adsorption with aminated poly(ethylene terephthalate) (APET) nanofiber mats.

The results for the kinetic experiments of Pb(II) adsorption are shown in Figure 4, demonstrating the adsorption capacity as a function of time. The results show a fast adsorption rate achieving a maximum adsorption capacity for Pb(II) over 30 min. Moreover, longer kinetics (until 240 min) provided similar adsorption capacities without significant variations. This fact demonstrates the likelihood of a strong interaction between the Pb(II) ions and the lone-pair nitrogen electrons of the primary amine group on the surface of the nanofibers. A maximum adsorption capacity test using Pb(II) mono-elemental solutions revealed a maximum adsorption capacity of 1.72 mmol Pb(II)/g APET after 30 min.

#### 3.3.2. Effect of pH on the Adsorption Process

**Figure 5:**
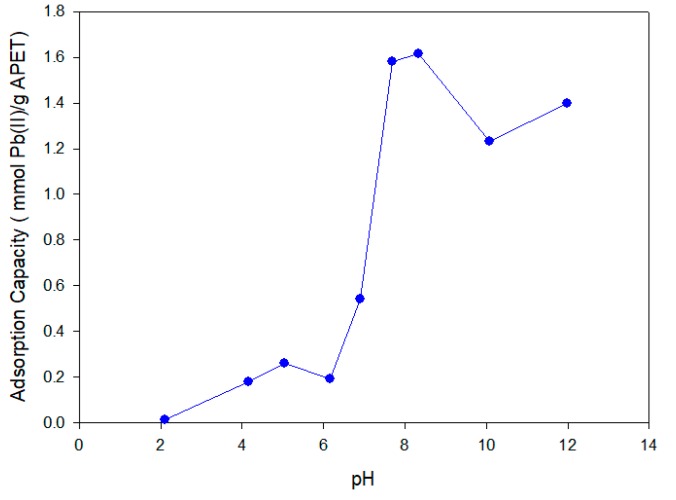
Effect of the initial pH on the Pb(II) adsorption with aminated poly(ethylene terephthalate) (APET) nanofiber mats.

The results of the pH-effect experiments for Pb(II) adsorption are shown in Figure 5. At pH 2.0, there was low Pb(II) adsorption (below 0.1 mmol Pb(II)/g APET) because of the competitive adsorption between the prevalently available H^+^ and the Pb(II) ions. At a lower pH, amino (–NH_2_) groups are inclined to bind with hydrogen ions to form –NH_3_^+^. However, more binding sites (–NH_2_) are available with increasing pH, leading to higher adsorption capacities [40]. A high increase of the adsorption capacity in the pH range between 6.0 and 8.0 achieved equilibrium at around pH 8.3. At pH 8.0, the competitive adsorption of the OH^−^ ions had less effect and no precipitation of Pb(II) was observed, reaching a maximum adsorption capacity of 1.62 mmol Pb(II)/g APET. Experimentally, Pb(II) precipitation was observed with a pH greater than or equal to 10, producing a decrease in the adsorption capacity. At a higher pH, the precipitation of metal ions appeared according to the solubility constant (*K_sp_*) of such compounds [40]. In order to analyze the samples correctly and to quantify the metal content adsorbed on the material, 10 mL of 1.0 M HNO_3_ was added to the suspension and the precipitate was dissolved. Then, we ensured that the quantification of the metal ions was complete and that the obtained results were completely reliable.

#### 3.3.3. Effect of Metal Concentration on the Adsorption Process

**Figure 6:**
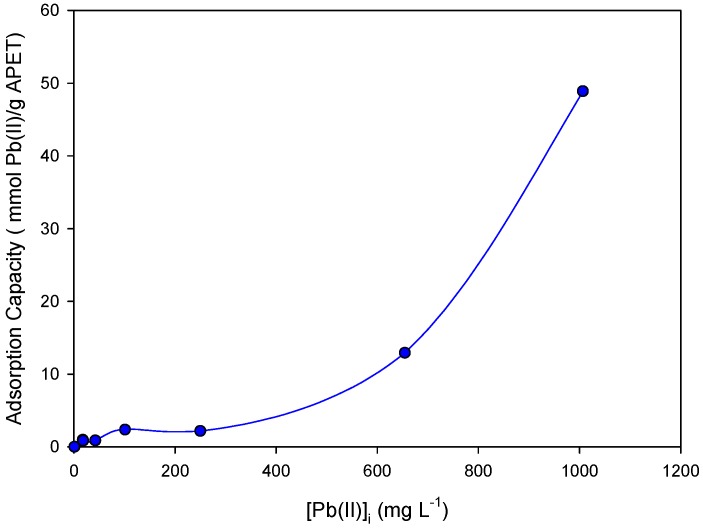
Effect of the initial concentration on the Pb(II) adsorption with aminated poly(ethylene terephthalate) (APET) nanofiber mats.

The effect of the Pb(II) initial concentration at optimal working conditions of the pH is shown in Figure 6. The adsorption capacity increased as a function of the Pb(II) concentration. At low initial Pb(II) concentrations, the adsorption capacity was low and revealed similar values until 200 mg L^−1^ was reached. As the concentration increased, the value of the adsorption capacity increased, providing a huge maximum adsorption capacity of 48.9 mmol Pb(II)/g APET at 1000 mg L^−1^.

The saturation point was not observed, and this could be because, at high concentrations, more Pb(II) ions are present in the solution that strengthen the APET nanofiber mats, thereby increasing the adsorption rate.

The metal uptake capacity of the developed APET nanofiber mats can be compared with values previously reported for PET-based nanofiber adsorbents [36,37,38,40]. Under similar experimental conditions (pH 6–8; contact time less than 60 min and initial metal concentration of 200–300 mg/L), the APET nanofibers showed adsorption capacities of up to 2.2 mmol Pb(II)/g (equivalent to 445.8 mg Pb(II)/g), which was 10 times higher than the maximum adsorption capacities reported for similar PET-based systems (45 mg Pb(II)/g).

#### 3.3.4. Selectivity towards Interfering Metal Ions

The selectivity towards the presence of metal ions was examined, and the results are shown in Table 4. The results indicate that, with multi-elemental solutions, competitive adsorption of Ni(II), Cu(II) and Cd(II) on APET nanofiber mats exists. As a consequence, the Pb(II) adsorption capacity decreased by 34% (in the molar ratio 1:1) and by 58% (in the molar ratio 1:2) compared with the mono-elemental solution.

The extent of such a reduction depends on the adsorption affinity of APET nanofibers to other metals. In spite of this, the experiments indicate that Pb(II) is the most-preferred metal ion. This fact could be explained on the basis of the higher stability of the complexes formed by Pb(II) ions with the amine groups of APET nanofiber mats. This stability might be dependent on the ionic radius and the ionic potential of the metal ions [47].

However, in spite of the reduction of the adsorption capacity for Pb(II), a strong increase in the uptake of Ni(II) was observed (8.3 mmol Ni(II)/g APET), demonstrating that APET nanofiber mats can be also applied to remove Ni(II) from contaminated water. Hence, we can conclude that under these conditions, the APET nanofiber mats show a strong preference for adsorbing both Pb(II) and Ni(II) over Cu(II) and Cd(II).

### 3.4. Adsorption Experiments in Continuous Mode

**Figure 7:**
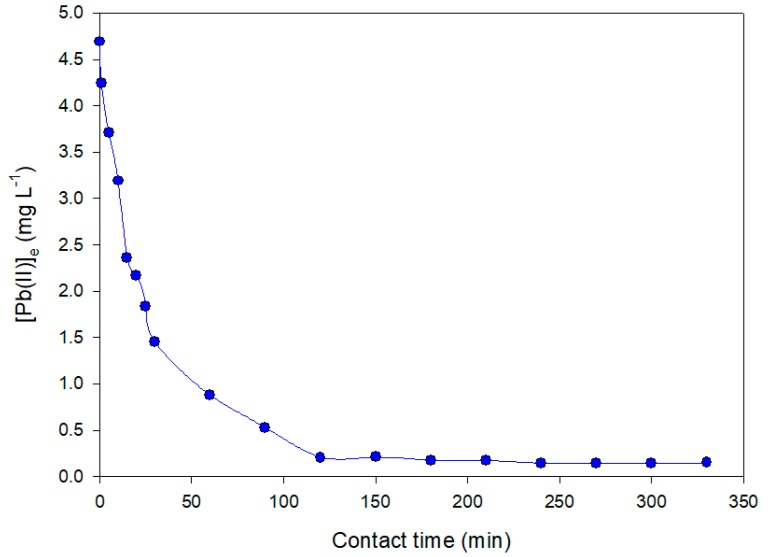
Pb(II) adsorption in continuous mode using aminated poly(ethylene terephthalate) (APET) nanofiber mats.

In order to prove the APET adsorption capacity in a continuous mode, a solution of 5 mg L^−1^ of Pb(II) was pumped throughout a multilayer cartridge of the APET nanofibers, achieving an adsorption of up to 97% of Pb(II) in the mono-elemental synthetic solution. The APET mats had the ability to extract virtually all of the Pb(II), showing a high reactivity of the nanofibers in the presence of Pb(II). The steady state was reached at around 240 min. The results in Figure 7 suggest that the APET nanofiber mats are a potential system for heavy metal removal from contaminated effluents.

## 5. Conclusions

In summary, an APET nanofibrous adsorbent system was developed by an electrospinning technique, followed by surface chemical modification of the electrospun PET nanofibers with EDA. EDA was demonstrated to be an adequate key reagent to provide stable primary amine groups over the PET nanofibers’ surface. The experimental conditions for the modification process were optimized, achieving a high functionalization degree.

The efficiency of APET nanofibers for Pb(II) adsorption was evaluated in batch mode by kinetic experiments. Under optimal conditions, the APET nanofiber mats presented a high interaction with Pb(II), reaching a huge maximum adsorption capacity of about 50 mmol of Pb(II) per gram of adsorbent system. Moreover, the APET nanofibers showed a high Pb(II) selectivity in the presence of interfering metal ions such as Cu(II) and Cd(II), whereas a high Ni(II) adsorption rate was observed. The APET nanofibers also demonstrated excellent performance in continuous-flow mode operation, retaining their excellent uptake capacity and reaching 97% metal removal after 120 min.

## Figures and Tables

**Table 1:** Electrospinning process parameters for obtaining poly(ethylene terephthalate) (PET) nanofibers.

Sample	PET Concentration (wt %)	TBAB Concentration (wt %)	Feed Rate (mL/h)	Voltage (kv)	Distance (mm)
PET-1	20	0.1	1.2	25	220
PET-2	15	0.1	1.0	28	180
PET- 3	15	0.2	0.5	30	150

**Table 2:** Properties of poly(ethylene terephthalate) (PET) solution and diameter of electrospun fibers.

Sample	PET (wt %)	TBAB (wt %)	Viscosity (cP)	Conductivity (µS/cm)	Fiber Diameter (nm)
PET-1	20	0.1	298.5	10.7	3000 ± 500
PET-2	15	0.1	170.4	46.8	724 ± 125
PET-3	15	0.2	174.5	126.2	488 ± 61

**Table 3:** Aminolysis conditions of the poly(ethylene terephthalate) (PET) nanofiber mats and elemental analysis for blank PET and aminated PET (APET).

Sample	EDA (wt %)	Time (min)	% N	% C	% H	% O	mg EDA/g
PET Blank	—	—	0.45	61.17	2.95	35.43	—
APET-1	10	60	0.92	60.84	3.25	34.99	0.02
APET-2		15	5.51	56.21	3.23	35.05	0.12
APET-3	5	60	0.90	60.68	3.35	35.08	0.02
APET-4		15	6.73	56.28	4.53	32.46	0.14
APET-5	2.5	60	0.86	61.63	2.73	34.78	0.02
APET-6		15	4.97	57.69	4.34	33.00	0.11

**Table 4:** Adsorption capacities (q) in multi-elemental solutions.

Pb(II)–Cd(II)/Ni(II)/Cu(II) Molar Ratio	*q* (Pb(II); mmol/g)	*q* (Ni(II); mmol/g)	*q* (Cu(II); mmol/g)	*q* (Cd(II); mmol/g)	Decrease of *q* (Pb(II); %)
1:0	1.78	—	—	—	—
1:1	1.17	0.11	0.45	0.28	34.27
1:2	0.74	8.26	0.65	1.62	58.43

## Supplementary Materials

The following are available online at www.mdpi.com/1996-1944/10/12/1352/s1. Figure S1: APET cartridge drawing for continuous adsorption experiments; Figure S2: Functionalization degree of PET nanofibers at room temperature; Figure S3: Kaiser test results of blank PET and APET nanofiber mats.
