# Supplementary material for: Aminated Polyethylene Terephthalate (PET) Nanofibers for the Selective Removal of Pb(II) from Polluted Water
Source: Materials (Basel). 2017 Nov 24;10(12):1352. doi: 10.3390/ma10121352 (PMC5744287; doi:10.3390/ma10121352)
Supplement: Supplementary file 1 [file materials-10-01352-s001.pdf]

materials

Aminated Polyethylene Terephthalate (PET) Nanofibers for the Selective Removal of Pb(II) from Polluted Water

Diego Morillo Martín [1], Mohamed Ahmed [1], Mónica Rodríguez [2], María A. García [2] and Mirko Faccini [1,*]

Supplementary Materials: The following are available online at www.mdpi.com/link, Figure S1: APET cartridge drawing for continuous adsorption experiments. Figure S2. Functionalization degree of PET nanofiber at room temperature. Figure S3. Kaiser Test results of blank PET and APET nanofiber mats.

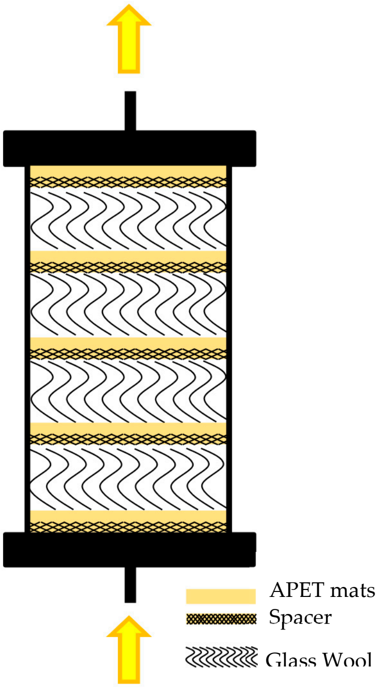

Figure S1. APET cartridge drawing for continuous adsorption experiments

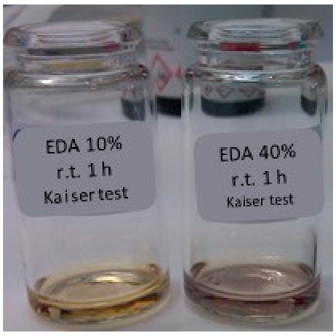

Figure S2. Functionalization degree of PET nanofiber at room temperature

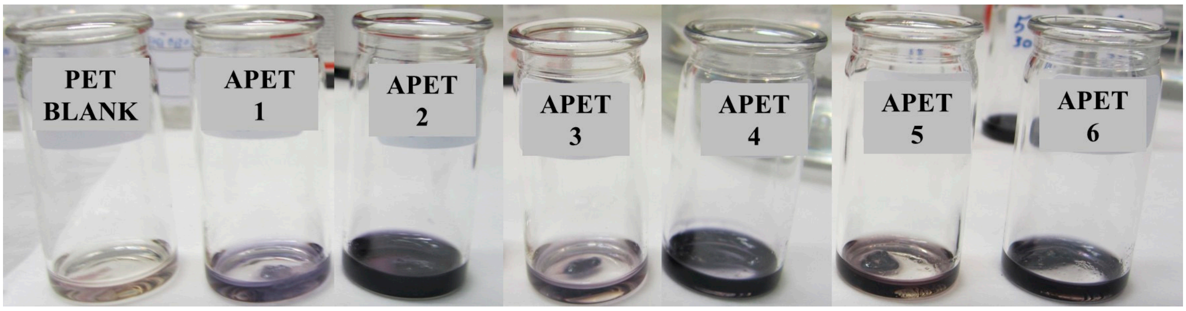

Figure S3. Kaiser Test results of blank PET and APET nanofiber mats
